# Age distribution types of bladder cancers and their relationship with opium consumption and smoking

**Published:** 2015

**Authors:** Arsalan Aliramaji, Aliakbar Kaseean, Yousef Reza Yousefnia Pasha, Hamid Shafi, Sekineh Kamali, Mohsen Safari, Emaduddin Moudi

**Affiliations:** 1 Department of Urology, Babol University of Medical Sciences, Babol, Iran.; 2 Clinical Research Development Center, Babol University of Medical Sciences, Babol, Iran.; 3 Babol University of Medical Sciences, Babol, Iran.

**Keywords:** Bladder cancer, Smoking, Opium.

## Abstract

**Background:**

Recognition of the predisposing factors of bladder cancer is very important and provides possible prevention measures. The aim of this study was to investigate the types, distribution of bladder tumors and their relationship with opium consumption and smoking in patients who referred to Shahid Beheshti Hospital, Babol, Iran.

**Methods:**

In this case-control study, all patients diagnosed with bladder cancer who underwent surgery during 2001-2012 were enrolled. The subjects of the control group were selected among the patients who underwent ERCP (endoscopic retrograde cholangiopancreatography) for gallstone and had no tumors and genitourinary problems. Data regarding demographic, pathology reports and tumor type, smoking status, history of opium consumption and its duration were collected. Patients and controls were compared using t-test and chi-square test. SPSS software Version 20 was used for analysis.

**Results:**

In this study, 175 patients with an average age of 63.30±15.29 years and 175 age- matched controls were studied. A significant association was observed between smoking and opium consumption with bladder cancer (P=0.001 for both).

**Conclusion:**

The results of this study showed that opium consumption and smoking are associated with bladder cancer

Bladder cancer is the fourth most common malignancy in men and the eighth in women in Iran (1, 2). According to the cancer division of the Center for Disease Control and Prevention (CDC) of the World Health Organization (WHO) (2005), bladder cancer (7.4%) is the most reported cancer in Iran (3-5). The incidence of bladder cancer in men is 2.5 to 4 times more than in women (6, 7). The average age of involvement is 69 years old and the disease is more common in whites (8, 9). 

More than 98% of bladder cancers are from epithelial origin, among which 92% of them is a transitional type (10). Initial symptoms in most patients who referred to the hospital are pain-less hematuria, dysuria and frequent urination (1, 11). The most important risk factors that have been reported so far are smoking (that doubles the risk of bladder cancer), and occupational exposures (2, 3, 12). 

The data regarding bladder cancer and opium consumption as well as smoking are rare. Our experience showed that the most diagnosed cases of bladder cancer in our hospital were opium users, so we performed the present study to investigate whether there is a relationship between these diseases and opium consumption.

## Methods

The study group consisted of patients diagnosed with bladder cancer who underwent a surgical operation during 2001-2012, and the specimen was sent for pathologic examination, cancer was confirmed. The total number of patients with bladder cancer was 236 people, but the number of patients excluded because of incomplete data was 61 cases, finally 175 patients were enrolled. The control group consisted of 175 patients, selected from patients who referred to the same hospital for the investigation of gall bladder stone. In addition, the control group had no genitourinary symptoms.

The subjects and the case group were matched in regard to age and sex. Patients with opium consumption or smoking of more than 1 year duration were included. All demographic data, pathology reports and bladder tumor type, history of smoking and opium consumption, and the duration in two groups were taken from the patients’ files in the Department of Pathology. Archives of Shahid Beheshti Hospital, Babol and via phone calls. All data were recorded in a checklist. In statistical analyses, the two groups were compared regarding smoking and opium consumption using chi square test. Furthermore, the relationship between opium or smoking with frequency and type of tumors were determined. SPSS Version 20, t-test, and X^2^ Fisher's exact test and logistic regression were used for the statistical analysis, p<0.05 was considered significant.

## Results

There were 350 people in the study, 175 patients were in the case group and 175 patients in the control group. 153 (87.4%) patients were males and 22 (12.6%) patients were females in both group. The mean age of patients was reported 62.81±16.21 in the range of 18-100 years in the case group and 63.78±14.35 years of age in the range of 34-91 years in the control group. In the case group, 19 patients (10.9%) were less than 40 years and 156 people (89.1%) above 40 years old. 

According to the pathology department for the case group, 168 (96%) patients suffered from transitional cell carcinoma (TCC), and other tumors included: adenocarcinoma in 5 cases (2.9%), squamous cell carcinoma (SCC) 1 case (0.57%), and liomyosarcoma 1 case (0%.57) (table 2).

In the case group, 44 patients consumed simultaneously both cigarette and opium, while in the control group, 20 patients from 175 patients consumed simultaneously cigarette and opium. Based on the statistical analysis, a significant association was observed between smoking and opium consumption increasing the risk of bladder cancer (P=0.001). In statistical analysis, opium consumption and smoking were significantly associated with bladder cancer (p<0.0001 for both (table 1). 

**Table 1:** Demographic characteristics of patients with bladder cancer and the control group 2001-2012

**Variable**	**Case group** **N(%)**	**Control group** **N(%)**
**Gender**		
Male Female	153 (87.4) 22 (12.6)	153 (87.4) 22 (12.6)
**Smoking**		
Yes No	94 (53.7 81 (46.3)	31 (17.7) 144 (82.3)
**Opium consumption**		
Yes No	58 (33.1) 117 (66.9)	27 (15.4) 148 (84.6)

**Figure 1:**
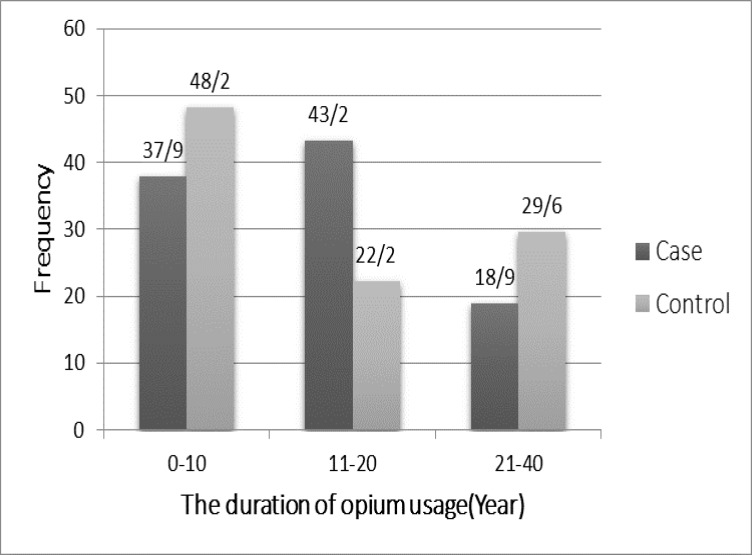
Duration of opium consumption in the study patients and controls 2001-2012

There was also a significant association between duration of opium consumption with bladder cancer (p<0.0001) (figure 1). 

Fifty–eight patients with bladder cancer were opium users with transitional cell carcinoma, and 3 patients (5.2%) were diagnosed with adenocarcinoma. 94 patients with bladder cancer were smokers, 90 patients (95.7%) of them had transitional cell carcinoma, 3 patients (3.2%) suffered from adenocarcinoma, and 1 patient (1.1%) had squamous cell carcinoma (**table 3**).

**Table 2:** Frequency of pathology according to age group

**Age group** **A variety of Pathology**	**18-39** **N(%)**	**40-60** **N(%)**	**61-80** **N(%)**	**81-100** **N(%)**
Transitional Cell Carcinoma	17(10.1)	43(25.6)	92(54.8)	16(9.5)
Adenocarcinoma	2(40)	2(40)	1(20)	-
Squamous cell Carcinoma	-	1(100)	-	-
Leiomyosarcoma	-	-	1(100)	-

## Discussion

Bladder cancer is the second most common malignancy of the genitourinary system worldwide (13). Identifying the predisposing and risk factors of the disease is of great importance and may provide disease prevention.

The results of the present study show that the male to female ratio is about 6.9 to 1. This ratio was 4 to 1 in Kerman province (14), and this is less than 3 in India, Thailand, and America, while in Southern Europe where smoking is higher, this ratio is more than 6 (15). This indicates that an increased risk of bladder cancer in men is more than in women due to occupational exposure and more exposure to cigarette smoking. 

In the present study, 10.9% of bladder cancer patients were younger than 40 years. In a study conducted in Spain (16), 2.95% of patients with bladder cancer were less than 40 years and this is not consistent with our results. In comparison with a previous study, the frequency of bladder cancer in our young patients was 3.5 times higher as in Spain (16). In the survey of age distribution of the study population showed that most people were in the age group of 61-80 years. In Yang’s (17) study (2013) in China and Akbarzadeh Pasha in Mazandaran province of Iran (2011) (18), the most frequency of bladder cancer was observed in the age group 60-79 and 70-84 years, respectively; which is consistent with our findings. In various studies, the average age of bladder cancer incidence was reported in 65-70 years (19). Increased engagement of women in industrial environments equal to men from 1985 reduced the age of the female patients (20). The mean age of patients with bladder cancer in this study was 62.81 years old. In a study conducted in Egypt (2009), the mean age at the time of diagnosis of bladder cancer was reported 60.9 years (21) which is similar to our study. The study conducted by Yang (2013) in China showed that the incidence of bladder cancer had an exact correlation with age. This means that with increasing age, the incidence of bladder cancer will increase (17). This trend was confirmed in epidemiological literatures (19). 

In epidemiological studies conducted in Kurdestan province, the frequency of transitional cell carcinoma, adenocarcinoma, and squamous were 97.9%, 1.6%, 0.5%, respectively; that is consistent with our results, and suggests that the incidence of TCC in Iran is more than the other parts of the world (22). 

In this study, by investing the frequency of smoking in the case group, it is resulted that smoking cigarettes definitely increases 4.86 times the risk of bladder cancer. While in the references, the risk of bladder cancer are 2 times more in smokers than others. These results indicate that the effect of smoking on the incidence of bladder cancer is more in our region. In a study by Asgari et al. (2004) conducted in Tehran, from the patients with bladder cancer, 68% were smokers while 23% patients in the control group were smokers, and it is stated that cigarette smoking will increase 3.8 times the risk of bladder cancer (10). It is more than the results that were obtained in our study.

According to the results of the study, in one-sample test of patients consume opium, the risk of bladder cancer increases as much as 2.71, while in the adjusted analysis, opium consumption does not have a significant impact. In a case-control study conducted by Nourbakhsh et al. (2006) in Tehran, 18% patients of the case group and 5% patients of the control group consumed opium. Thus, it was stated that opium consumption could increase 3.8 times the risk of bladder cancer (23). In another study by Ketanchi (2005) in Kerman, the consumption of opium was also known as the risk of bladder cancer (14). By summarizing these studies, it can clearly be seen that there is an association between opium consumption with bladder cancer in our country. 

So there is a particular concern to this group, recognizing the risk factors of the disease and providing appropriate controlling and managing measures in order to prevent the increasing incidence and early diagnosis of the disease are recommended. 

In conclusion, the results of this study showed that there is a relationship between the consumption of opium and bladder cancer. 

## Notes

**Funding:** This project was financially supported by the Research Chancellery of Babol University of Medical Sciences (Grant Number: 1741)
